# Dimethyl 4-(4-hy­droxy­phen­yl)-2,6-dimethyl-1,4-dihydro­pyridine-3,5-dicarboxyl­ate

**DOI:** 10.1107/S1600536811032521

**Published:** 2011-08-17

**Authors:** Chun-Hua Zhang, Jing-Min Zhao, Bao-Guo Chen

**Affiliations:** a College of Chemistry and Chemical Engineering, Inner Mongolia University for the Nationalities, Inner Mongolia Autonomous Region Tongliao, Huolinhe Street #22, 028000, People’s Republic of China; b Institute of Higher Vocational Education, Tongliao Vocational College, Inner Mongolia Autonomous Region Tongliao, Huolinhe Street #152, 028000, People’s Republic of China

## Abstract

The title mol­ecule, C_17_H_19_NO_5_, was prepared by a Hantzsch dihydro­pyridine synthesis from 4-hy­droxy­benzaldehyde, methyl acetoacetate and NH_4_HCO_3_. In the mol­ecular structure of the title compound, the dihydro­pyridine ring adopts a flattened boat conformation and the plane of the base of the boat forms a dihedral angle of 80.8 (2)° with the aromatic six-membered ring. The packing is stabilized by strong inter­molecular N—H⋯O_carbon­yl_, O_hydrox­y_—H⋯O_carbon­yl_ and weak intra­molecular C—H⋯O hydrogen bonds.

## Related literature

For background to the bioactivity and synthesis of 1,4-dihydro­pyridines, see: Yang *et al.* (2010 ▶); Davies *et al.* (2005 ▶); Warrior *et al.* (2005 ▶); Ko & Yao (2006 ▶); Rose & Draeger (1992 ▶). For related structures, see: Bai *et al.* (2009 ▶); Fun *et al.* (2009 ▶); Thenmozhi *et al.* (2009 ▶). For hydrogen-bond definitions, see: Desiraju & Steiner (1999 ▶). For the synthetic method, see: Tamaddon *et al.* (2010 ▶).
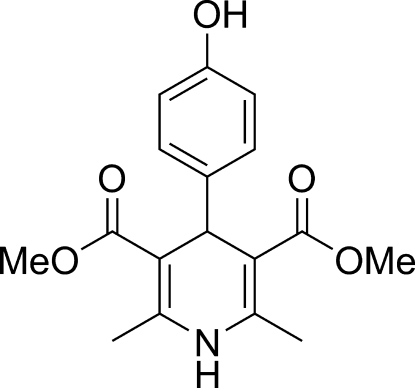


## Experimental

### 

#### Crystal data

C_17_H_19_NO_5_
*M* *_r_* = 317.33
Monoclinic, 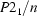

*a* = 13.245 (3) Å
*b* = 9.3480 (19) Å
*c* = 13.754 (3) Å
β = 110.14 (3)°
*V* = 1598.8 (6) Å^3^
*Z* = 4
Mo *K*α radiation
μ = 0.10 mm^−1^
*T* = 293 K
0.20 × 0.10 × 0.10 mm

#### Data collection

Nonius CAD-4 diffractometer
Absorption correction: ψ scan For semi-empirical (using intensity measurements) absorption, see: North *et al.* (1968 ▶) *T*_min_ = 0.981, *T*_max_ = 0.990
4588 measured reflections
2931 independent reflections
1212 reflections with *I* > 2σ(*I*)
*R*_int_ = 0.104
3 standard reflections every 200 reflections  intensity decay: 1%

#### Refinement

*R*[*F* ^2^ > 2σ(*F* ^2^)] = 0.074
*wR*(*F* ^2^) = 0.081
*S* = 1.00
2931 reflections
209 parameters
1 restraint
H-atom parameters constrained
Δρ_max_ = 0.16 e Å^−3^
Δρ_min_ = −0.17 e Å^−3^

### 

Data collection: *CAD-4 EXPRESS* (Enraf–Nonius, 1994 ▶); cell refinement: *CAD-4 EXPRESS*; data reduction: *XCAD4* (Harms & Wocadlo, 1996 ▶); program(s) used to solve structure: *SHELXS97* (Sheldrick, 2008 ▶); program(s) used to refine structure: *SHELXL97* (Sheldrick, 2008 ▶); molecular graphics: *SHELXTL* (Sheldrick, 2008 ▶); software used to prepare material for publication: *SHELXTL*.

## Supplementary Material

Crystal structure: contains datablock(s) I, global. DOI: 10.1107/S1600536811032521/zl2397sup1.cif

Structure factors: contains datablock(s) I. DOI: 10.1107/S1600536811032521/zl2397Isup2.hkl

Supplementary material file. DOI: 10.1107/S1600536811032521/zl2397Isup3.mol

Supplementary material file. DOI: 10.1107/S1600536811032521/zl2397Isup4.cml

Additional supplementary materials:  crystallographic information; 3D view; checkCIF report

## Figures and Tables

**Table 1:** Hydrogen-bond geometry (Å, °)

*D*—H⋯*A*	*D*—H	H⋯*A*	*D*⋯*A*	*D*—H⋯*A*
N1—H1*A*⋯O4^i^	0.86	2.10	2.936 (4)	164
O1—H1*B*⋯O2^ii^	0.82	1.92	2.742 (4)	179
C7—H7*A*⋯O2	0.98	2.39	2.781 (5)	103
C7—H7*A*⋯O5	0.98	2.32	2.717 (5)	103
C12—H12*A*⋯O3	0.96	2.06	2.790 (5)	131
C13—H13*A*⋯O4	0.96	2.26	2.818 (5)	116

Symmetry codes: (i) 
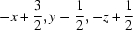
; (ii) 
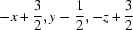
.

**Fig. 1.**
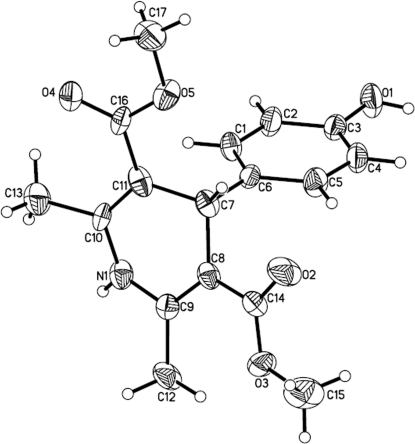
Molecular structure of the title compound, showing the atom-numbering scheme. Displacement ellipsoids are drawn at the 50% probability level.

**Fig. 2.**
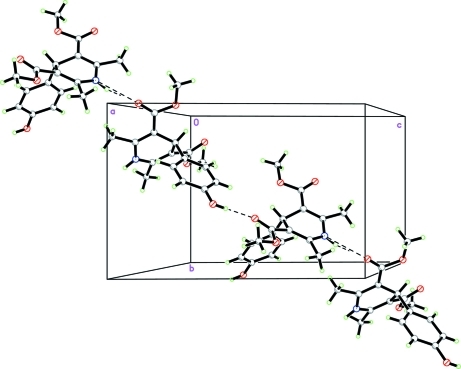
The packing of the title compound, viewed along the a axis. Dashed lines indicate hydrogen bonds.

## supplementary crystallographic information

### Comment

In recent years, much attention has been focused on the synthesis of 1,4-dihydropyridine derivatives because of their presence in numerous natural products along with a wide spectrum of their physiological activities (Yang *et al.*, 2010). For example, some dihydropyridines have calcium modulatory properties (Rose & Draeger, 1992), antibacterial activity (Davies *et al.*, 2005), or fungicidal activity (Warrior *et al.*, 2005), to name just a few. Because of the biological importance associated with these compounds, numerous methods had been developed for the synthesis of 1,4-dihydropyridine derivatives, which include the use of microwaves, ionic liquids, refluxing at high temperature, metal triflates, and iodine (Ko & Yao, 2006). However, the use of high temperatures, expensive metal precursors, catalysts that are harmful to the environment, and long reaction times limit the use of many of these methods. Herein, we report a mild and catalyst-free synthesis based on a variation of the commonly used Hantzsch dihydropyridine synthesis (Tamaddon *et al.*, 2010), and the crystal structure of the resultant title compound is presented. Compared to the classical method involving the three-component coupling of an aldehyde with ethyl acetoacetate, and ammonia in acetic acid or in refluxing alcohol, the reation was conducted in water and  avoided the use of catalysts, so it was very environmentally benign. Moreover, the workup is very simple and can give the product in high yield after simple filtration.

In the molecular structure of the title compound (Fig. 1), atoms C7 and N1 deviate from the mean plane of atoms C8/C9/C10/C11 in the same direction, by 0.45 (0) and 0.18 (7) Å, respectively, so the heterocyclic ring adopts a boat conformation. In addition, the phenol ring substituent is almost perpendicular to the plane of the atoms C8/C9/C10/C11, with a dihedral angle between them of 80.8 (2)°. The methyl groups are nearly coplanar with the aformentioned plane of the atoms C8/C9/C10/C11, with the methyl C atoms C12 and C13 deviating from the mean plane by 0.12 (4) and 0.22 (2) Å, respectively. The average C—N bond lengths of the title compound are with 1.353 (9) Å similar to those of its phenyl substituted 1,4-dihydropyridine derivative (Bai *et al.*, 2009) which has average C—N bond lenghts of 1.376 (8) Å, its 4-methoxyphenyl substituted 1,4-dihydropyridine derivative (Thenmozhi *et al.*, 2009) which has average C—N bond lenghts of 1.377 (4) Å, and its 4-methylphenyl substituted 1,4-dihydropyridine derivative (Fun *et al.*, 2009) which has average C—N bond lenghts of 1.385 (7) Å.

The crystal packing of the title compound is stabilized by strong intermolecular N—H···O_carbonyl_ and O_hydroxyl_—H···O_carbonyl_ hydrogen bonds (Desiraju & Steiner, 1999), N1—H1A···O4^i^ and O1—H1B···O2^ii^, and by several weaker intramolecular C—H···O hydrogen bonds, C7—H7A···O2, C7–H7A···O5, C12—H12A···O3, C13—H13A···O4 (see Table 1 for numerical values and symmetry operators).

### Experimental

The title compound was obtained according to a reported method (Tamaddon *et al.*, 2010). A mixture of 4-hydroxybenzaldehyde (2 mmol), methyl acetoacetate (4 mmol), and NH_4_HCO_3_ (2 mmol) was stirred in water (2 mL) under reflux. After completion of the reaction (TLC monitoring), the mixture was diluted with cold water (20 mL) and filtered to obtain the precipitated product which was further purified by recrystallization. Single crystals suitable for X-ray diffraction were obtained by slow evaporation of an ethanol solution. IR (KBr) *v*/cm^-1^: 3327, 2960, 1685, 1652; ^1^H NMR (300 MHz, DMSO-*d6*) *δ*/ppm: 9.05 (s, 1H, NH), 8.77 (s, 1H, OH), 6.91 (d, 2H, ArH, J = 7.8 Hz), 6.56 (d, 2H, ArH, J = 7.8 Hz), 4.77 (s, 1H, H4), 3.55 (s, 6H, 2COOCH_3_), 2.25 (s, 6H, 2CH_3_); MS (ESI) m/z: 318.1 [M+H]^+^, 340.1 [M+Na]^+^, 356.0 [M+K]^+^; Anal. Calcd for C_17_H_19_NO_5_: C, 64.34; H, 6.03; N, 4.41; found: C, 64.46; H, 6.09; N, 4.33.

### Refinement

All H atoms were located in a difference map and refined isotropically. The N-H distance of H1A atom (for N1) was constrained to 0.86 Å. All other H atoms were positioned geometrically and treated as riding, with C-H distances in the range 0.93-0.98 Å, an O-H distance of 0.82 Å and U_iso_(H) = 1.2 or 1.5 times U_eq_(C). The methyl groups were allowed to rotate during the refinement.

### Crystal data

**Table:**

C_17_H_19_NO_5_	*F*(000) = 672
*M**_r_* = 317.33	*D*_x_ = 1.318 Mg m^−^^3^
Monoclinic, *P*2_1_/*n*	Mo *K*α radiation, λ = 0.71073 Å
Hall symbol: -P 2yn	Cell parameters from 25 reflections
*a* = 13.245 (3) Å	θ = 9–12°
*b* = 9.3480 (19) Å	µ = 0.10 mm^−^^1^
*c* = 13.754 (3) Å	*T* = 293 K
β = 110.14 (3)°	Block, yellow
*V* = 1598.8 (6) Å^3^	0.20 × 0.10 × 0.10 mm
*Z* = 4	

### Data collection

**Table:**

Nonius CAD 4 diffractometer	1212 reflections with *I* > 2σ(*I*)
Radiation source: fine-focus sealed tube	*R*_int_ = 0.104
graphite	θ_max_ = 25.4°, θ_min_ = 1.8°
ω/2θ scans	*h* = 0→15
Absorption correction: ψ scan For semi-empirical (using intensity measurements) absorption, see: North *et al.* (1968)	*k* = −4→11
*T*_min_ = 0.981, *T*_max_ = 0.990	*l* = −16→15
4588 measured reflections	3 standard reflections every 200 reflections
2931 independent reflections	intensity decay: 1%

### Refinement

**Table:**

Refinement on *F*^2^	Primary atom site location: structure-invariant direct methods
Least-squares matrix: full	Secondary atom site location: difference Fourier map
*R*[*F*^2^ > 2σ(*F*^2^)] = 0.074	Hydrogen site location: inferred from neighbouring sites
*wR*(*F*^2^) = 0.081	H-atom parameters constrained
*S* = 1.00	*w* = 1/[σ^2^(*F*_o_^2^) + (0.005*P*)^2^] where *P* = (*F*_o_^2^ + 2*F*_c_^2^)/3
2931 reflections	(Δ/σ)_max_ < 0.001
209 parameters	Δρ_max_ = 0.16 e Å^−^^3^
1 restraint	Δρ_min_ = −0.17 e Å^−^^3^

### Special details

**Table:**

Geometry. All esds (except the esd in the dihedral angle between two l.s. planes) are estimated using the full covariance matrix. The cell esds are taken into account individually in the estimation of esds in distances, angles and torsion angles; correlations between esds in cell parameters are only used when they are defined by crystal symmetry. An approximate (isotropic) treatment of cell esds is used for estimating esds involving l.s. planes.
Refinement. Refinement of *F*^2^ against ALL reflections. The weighted *R*-factor *wR* and goodness of fit *S* are based on *F*^2^, conventional *R*-factors *R* are based on *F*, with *F* set to zero for negative *F*^2^. The threshold expression of *F*^2^ > σ(*F*^2^) is used only for calculating *R*-factors(gt) *etc*. and is not relevant to the choice of reflections for refinement. *R*-factors based on *F*^2^ are statistically about twice as large as those based on *F*, and *R*- factors based on ALL data will be even larger.

### Fractional atomic coordinates and isotropic or equivalent isotropic displacement parameters (Å^2^)

**Table:**

	*x*	*y*	*z*	*U*_iso_*/*U*_eq_	
N1	0.6521 (3)	0.2025 (3)	0.3016 (2)	0.0547 (9)	
H1A	0.6515	0.1445	0.2529	0.066*	
O1	1.0141 (2)	−0.0866 (3)	0.7601 (2)	0.0705 (9)	
H1B	0.9967	−0.1157	0.8083	0.106*	
C1	0.8797 (3)	0.1492 (4)	0.5474 (3)	0.0460 (10)	
H1C	0.8910	0.1793	0.4875	0.055*	
O2	0.5444 (2)	0.3203 (4)	0.5778 (2)	0.0866 (11)	
C2	0.9528 (3)	0.0542 (4)	0.6113 (3)	0.0609 (12)	
H2A	1.0116	0.0232	0.5947	0.073*	
O3	0.4353 (2)	0.1763 (3)	0.4605 (2)	0.0698 (9)	
C3	0.9371 (3)	0.0055 (4)	0.7006 (3)	0.0498 (10)	
O4	0.8684 (2)	0.5538 (3)	0.3923 (2)	0.0706 (9)	
C4	0.8511 (3)	0.0495 (4)	0.7228 (3)	0.0601 (12)	
H4A	0.8396	0.0157	0.7816	0.072*	
O5	0.8363 (2)	0.5462 (3)	0.5423 (2)	0.0600 (8)	
C5	0.7786 (3)	0.1468 (4)	0.6570 (3)	0.0578 (12)	
H5A	0.7195	0.1763	0.6735	0.069*	
C6	0.7922 (3)	0.2018 (4)	0.5662 (3)	0.0422 (9)	
C7	0.7113 (3)	0.3079 (4)	0.4988 (3)	0.0466 (9)	
H7A	0.7036	0.3853	0.5438	0.056*	
C8	0.5986 (3)	0.2431 (4)	0.4446 (3)	0.0480 (10)	
C9	0.5808 (3)	0.1810 (4)	0.3502 (3)	0.0442 (9)	
C10	0.7253 (3)	0.3108 (4)	0.3254 (3)	0.0461 (10)	
C11	0.7525 (3)	0.3737 (4)	0.4184 (3)	0.0530 (11)	
C12	0.4874 (3)	0.0857 (4)	0.2903 (3)	0.0674 (14)	
H12A	0.4384	0.0774	0.3276	0.101*	
H12B	0.5139	−0.0073	0.2819	0.101*	
H12C	0.4508	0.1269	0.2234	0.101*	
C13	0.7703 (3)	0.3459 (5)	0.2409 (3)	0.0789 (16)	
H13A	0.8426	0.3807	0.2715	0.118*	
H13B	0.7266	0.4180	0.1962	0.118*	
H13C	0.7703	0.2613	0.2013	0.118*	
C14	0.5229 (3)	0.2511 (4)	0.4999 (3)	0.0518 (11)	
C15	0.3573 (3)	0.1894 (5)	0.5099 (3)	0.1006 (19)	
H15A	0.2957	0.1319	0.4740	0.151*	
H15B	0.3359	0.2877	0.5088	0.151*	
H15C	0.3878	0.1575	0.5804	0.151*	
C16	0.8259 (3)	0.4957 (4)	0.4461 (3)	0.0506 (10)	
C17	0.9061 (3)	0.6675 (4)	0.5735 (3)	0.0797 (15)	
H17A	0.9123	0.6948	0.6426	0.120*	
H17B	0.8770	0.7457	0.5271	0.120*	
H17C	0.9759	0.6433	0.5717	0.120*	

### Atomic displacement parameters (Å^2^)

**Table:**

	*U*^11^	*U*^22^	*U*^33^	*U*^12^	*U*^13^	*U*^23^
N1	0.077 (3)	0.042 (2)	0.048 (2)	0.0006 (19)	0.0258 (18)	0.0007 (17)
O1	0.074 (2)	0.085 (2)	0.0554 (19)	0.0210 (18)	0.0265 (16)	0.0311 (17)
C1	0.060 (3)	0.049 (3)	0.036 (2)	0.010 (2)	0.0251 (19)	−0.0016 (17)
O2	0.075 (2)	0.127 (3)	0.059 (2)	−0.018 (2)	0.0253 (17)	−0.038 (2)
C2	0.058 (3)	0.073 (3)	0.057 (3)	0.004 (2)	0.027 (2)	0.024 (2)
O3	0.0503 (18)	0.104 (3)	0.063 (2)	−0.0190 (18)	0.0301 (16)	−0.0103 (19)
C3	0.056 (3)	0.053 (2)	0.045 (2)	−0.006 (2)	0.024 (2)	0.001 (2)
O4	0.085 (2)	0.075 (2)	0.0512 (19)	−0.0162 (18)	0.0227 (16)	0.0111 (16)
C4	0.066 (3)	0.075 (3)	0.045 (3)	0.003 (3)	0.026 (2)	0.014 (2)
O5	0.087 (2)	0.0338 (16)	0.071 (2)	−0.0074 (15)	0.0426 (18)	−0.0083 (15)
C5	0.052 (3)	0.075 (3)	0.054 (3)	0.011 (2)	0.029 (2)	0.007 (2)
C6	0.048 (2)	0.042 (2)	0.041 (2)	−0.0051 (18)	0.0206 (19)	0.0032 (17)
C7	0.054 (3)	0.049 (2)	0.039 (2)	0.012 (2)	0.0183 (19)	−0.0042 (19)
C8	0.065 (3)	0.042 (2)	0.036 (2)	0.0020 (19)	0.015 (2)	−0.0039 (17)
C9	0.042 (2)	0.045 (2)	0.047 (2)	0.0111 (19)	0.0175 (18)	0.007 (2)
C10	0.058 (3)	0.046 (2)	0.046 (2)	0.000 (2)	0.032 (2)	0.0108 (19)
C11	0.077 (3)	0.043 (2)	0.037 (2)	0.014 (2)	0.019 (2)	−0.0060 (19)
C12	0.064 (3)	0.097 (4)	0.040 (2)	−0.004 (3)	0.016 (2)	−0.021 (3)
C13	0.086 (4)	0.103 (4)	0.054 (3)	−0.005 (3)	0.032 (3)	−0.003 (3)
C14	0.051 (3)	0.059 (3)	0.040 (2)	0.002 (2)	0.009 (2)	−0.002 (2)
C15	0.055 (3)	0.136 (5)	0.112 (4)	0.008 (4)	0.031 (3)	−0.018 (4)
C16	0.059 (3)	0.049 (3)	0.053 (3)	0.002 (2)	0.031 (2)	0.016 (2)
C17	0.096 (4)	0.052 (3)	0.075 (3)	−0.008 (3)	0.009 (3)	0.000 (3)

### Geometric parameters (Å, °)

**Table:**

N1—C9	1.346 (4)	C7—C11	1.522 (4)
N1—C10	1.361 (4)	C7—C8	1.544 (5)
N1—H1A	0.8600	C7—H7A	0.9800
O1—C3	1.370 (4)	C8—C9	1.367 (4)
O1—H1B	0.8200	C8—C14	1.455 (5)
C1—C6	1.363 (4)	C9—C12	1.517 (4)
C1—C2	1.383 (4)	C10—C11	1.340 (5)
C1—H1C	0.9300	C10—C13	1.514 (4)
O2—C14	1.199 (4)	C11—C16	1.461 (5)
C2—C3	1.391 (4)	C12—H12A	0.9600
C2—H2A	0.9300	C12—H12B	0.9600
O3—C14	1.302 (4)	C12—H12C	0.9600
O3—C15	1.424 (4)	C13—H13A	0.9600
C3—C4	1.341 (4)	C13—H13B	0.9600
O4—C16	1.203 (4)	C13—H13C	0.9600
C4—C5	1.403 (5)	C15—H15A	0.9600
C4—H4A	0.9300	C15—H15B	0.9600
O5—C16	1.366 (4)	C15—H15C	0.9600
O5—C17	1.433 (4)	C17—H17A	0.9600
C5—C6	1.417 (4)	C17—H17B	0.9600
C5—H5A	0.9300	C17—H17C	0.9600
C6—C7	1.520 (5)		
			
C9—N1—C10	123.7 (3)	C11—C10—N1	119.4 (3)
C9—N1—H1A	118.2	C11—C10—C13	126.2 (4)
C10—N1—H1A	118.2	N1—C10—C13	114.4 (4)
C3—O1—H1B	109.5	C10—C11—C16	121.8 (4)
C6—C1—C2	124.5 (3)	C10—C11—C7	118.1 (4)
C6—C1—H1C	117.8	C16—C11—C7	119.9 (3)
C2—C1—H1C	117.8	C9—C12—H12A	109.5
C1—C2—C3	119.1 (4)	C9—C12—H12B	109.5
C1—C2—H2A	120.5	H12A—C12—H12B	109.5
C3—C2—H2A	120.5	C9—C12—H12C	109.5
C14—O3—C15	116.3 (3)	H12A—C12—H12C	109.5
C4—C3—O1	124.9 (4)	H12B—C12—H12C	109.5
C4—C3—C2	119.8 (4)	C10—C13—H13A	109.5
O1—C3—C2	115.2 (3)	C10—C13—H13B	109.5
C3—C4—C5	119.9 (4)	H13A—C13—H13B	109.5
C3—C4—H4A	120.1	C10—C13—H13C	109.5
C5—C4—H4A	120.1	H13A—C13—H13C	109.5
C16—O5—C17	113.8 (3)	H13B—C13—H13C	109.5
C4—C5—C6	122.4 (4)	O2—C14—O3	124.5 (4)
C4—C5—H5A	118.8	O2—C14—C8	120.0 (4)
C6—C5—H5A	118.8	O3—C14—C8	115.5 (4)
C1—C6—C5	114.3 (4)	O3—C15—H15A	109.5
C1—C6—C7	126.0 (3)	O3—C15—H15B	109.5
C5—C6—C7	119.8 (3)	H15A—C15—H15B	109.5
C6—C7—C11	110.6 (3)	O3—C15—H15C	109.5
C6—C7—C8	113.6 (3)	H15A—C15—H15C	109.5
C11—C7—C8	109.7 (3)	H15B—C15—H15C	109.5
C6—C7—H7A	107.6	O4—C16—O5	121.8 (4)
C11—C7—H7A	107.6	O4—C16—C11	127.0 (4)
C8—C7—H7A	107.6	O5—C16—C11	111.0 (3)
C9—C8—C14	126.5 (4)	O5—C17—H17A	109.5
C9—C8—C7	116.5 (3)	O5—C17—H17B	109.5
C14—C8—C7	117.0 (3)	H17A—C17—H17B	109.5
N1—C9—C8	119.2 (4)	O5—C17—H17C	109.5
N1—C9—C12	113.5 (3)	H17A—C17—H17C	109.5
C8—C9—C12	127.3 (3)	H17B—C17—H17C	109.5
			
C6—C1—C2—C3	1.0 (7)	C7—C8—C9—C12	167.3 (4)
C1—C2—C3—C4	0.9 (7)	C9—N1—C10—C11	21.1 (6)
C1—C2—C3—O1	−179.1 (4)	C9—N1—C10—C13	−160.3 (3)
O1—C3—C4—C5	178.6 (4)	N1—C10—C11—C16	−176.5 (3)
C2—C3—C4—C5	−1.3 (7)	C13—C10—C11—C16	5.0 (7)
C3—C4—C5—C6	0.0 (7)	N1—C10—C11—C7	7.9 (6)
C2—C1—C6—C5	−2.1 (6)	C13—C10—C11—C7	−170.6 (4)
C2—C1—C6—C7	179.0 (4)	C6—C7—C11—C10	92.2 (4)
C4—C5—C6—C1	1.6 (6)	C8—C7—C11—C10	−33.9 (5)
C4—C5—C6—C7	−179.4 (4)	C6—C7—C11—C16	−83.5 (4)
C1—C6—C7—C11	−12.1 (5)	C8—C7—C11—C16	150.4 (3)
C5—C6—C7—C11	169.1 (3)	C15—O3—C14—O2	5.5 (7)
C1—C6—C7—C8	111.8 (4)	C15—O3—C14—C8	−176.4 (4)
C5—C6—C7—C8	−67.0 (4)	C9—C8—C14—O2	−172.9 (4)
C6—C7—C8—C9	−88.5 (4)	C7—C8—C14—O2	8.7 (6)
C11—C7—C8—C9	35.9 (5)	C9—C8—C14—O3	8.9 (6)
C6—C7—C8—C14	90.1 (4)	C7—C8—C14—O3	−169.5 (4)
C11—C7—C8—C14	−145.5 (3)	C17—O5—C16—O4	−3.2 (6)
C10—N1—C9—C8	−18.3 (6)	C17—O5—C16—C11	−179.1 (3)
C10—N1—C9—C12	162.1 (4)	C10—C11—C16—O4	0.8 (7)
C14—C8—C9—N1	169.3 (4)	C7—C11—C16—O4	176.3 (4)
C7—C8—C9—N1	−12.3 (5)	C10—C11—C16—O5	176.4 (4)
C14—C8—C9—C12	−11.2 (7)	C7—C11—C16—O5	−8.0 (5)

### Hydrogen-bond geometry (Å, °)

**Table:**

*D*—H···*A*	*D*—H	H···*A*	*D*···*A*	*D*—H···*A*
N1—H1A···O4^i^	0.86	2.10	2.936 (4)	164
O1—H1B···O2^ii^	0.82	1.92	2.742 (4)	179
C7—H7A···O2	0.98	2.39	2.781 (5)	103
C7—H7A···O5	0.98	2.32	2.717 (5)	103
C12—H12A···O3	0.96	2.06	2.790 (5)	131
C13—H13A···O4	0.96	2.26	2.818 (5)	116

Symmetry codes: (i) −*x*+3/2, *y*−1/2, −*z*+1/2; (ii) −*x*+3/2, *y*−1/2, −*z*+3/2.
